# Supplementary figures and images for: Fat body-specific reduction of CTPS alleviates HFD-induced obesity
Source: eLife. 2023 Sep 11;12:e85293. doi: 10.7554/eLife.85293 (PMC10495109; doi:10.7554/eLife.85293)

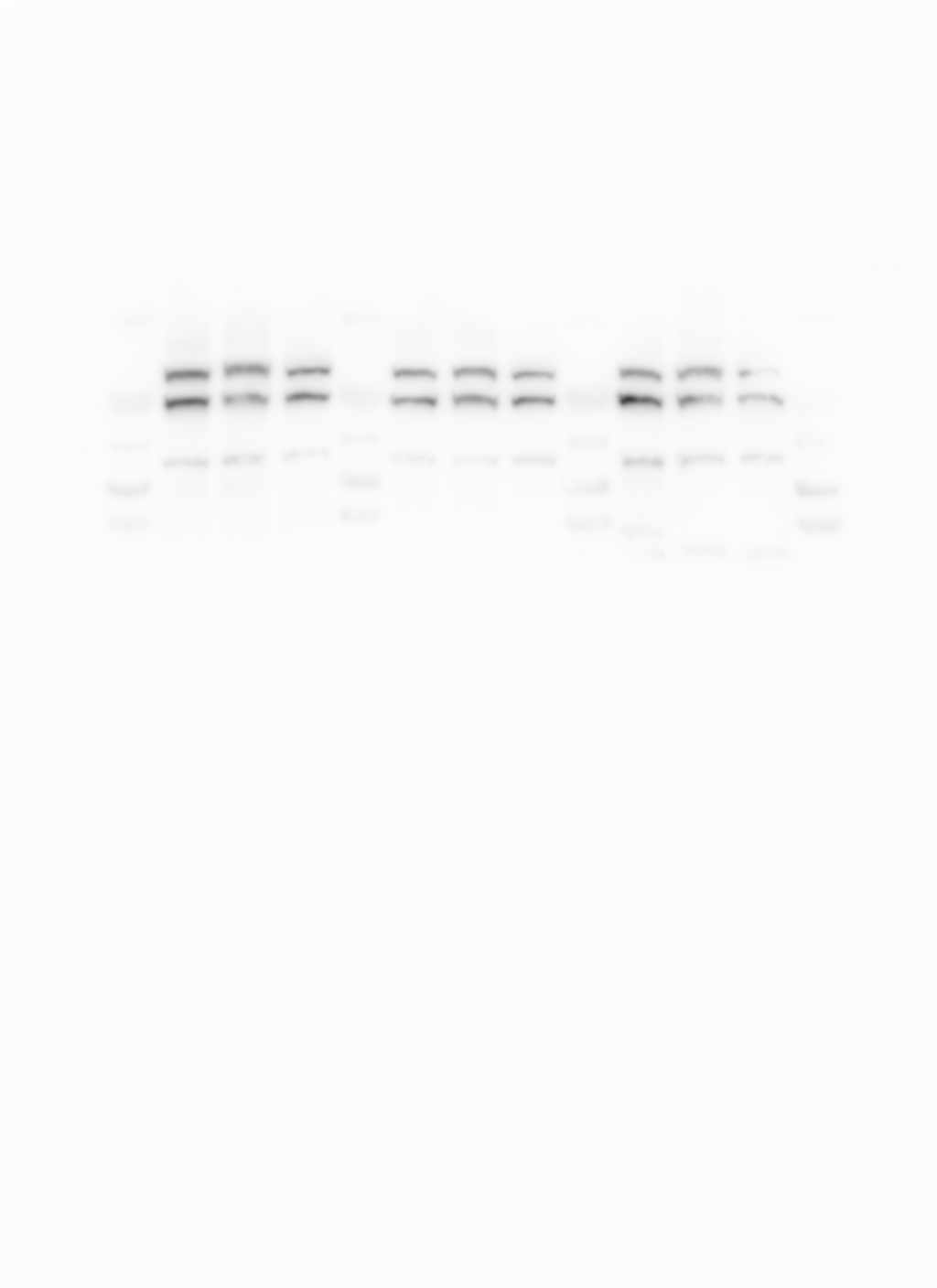

Supplement: Figure 5—source data 2. — Anti-mCh, anti-phosphorylated-Akt, and anti-total-Akt antibodies were used for the immunoblotting analysis. Alpha-tubulin was used as an internal control. [file elife-85293-fig5-data2.zip › Figure 5-source data 2.tif]

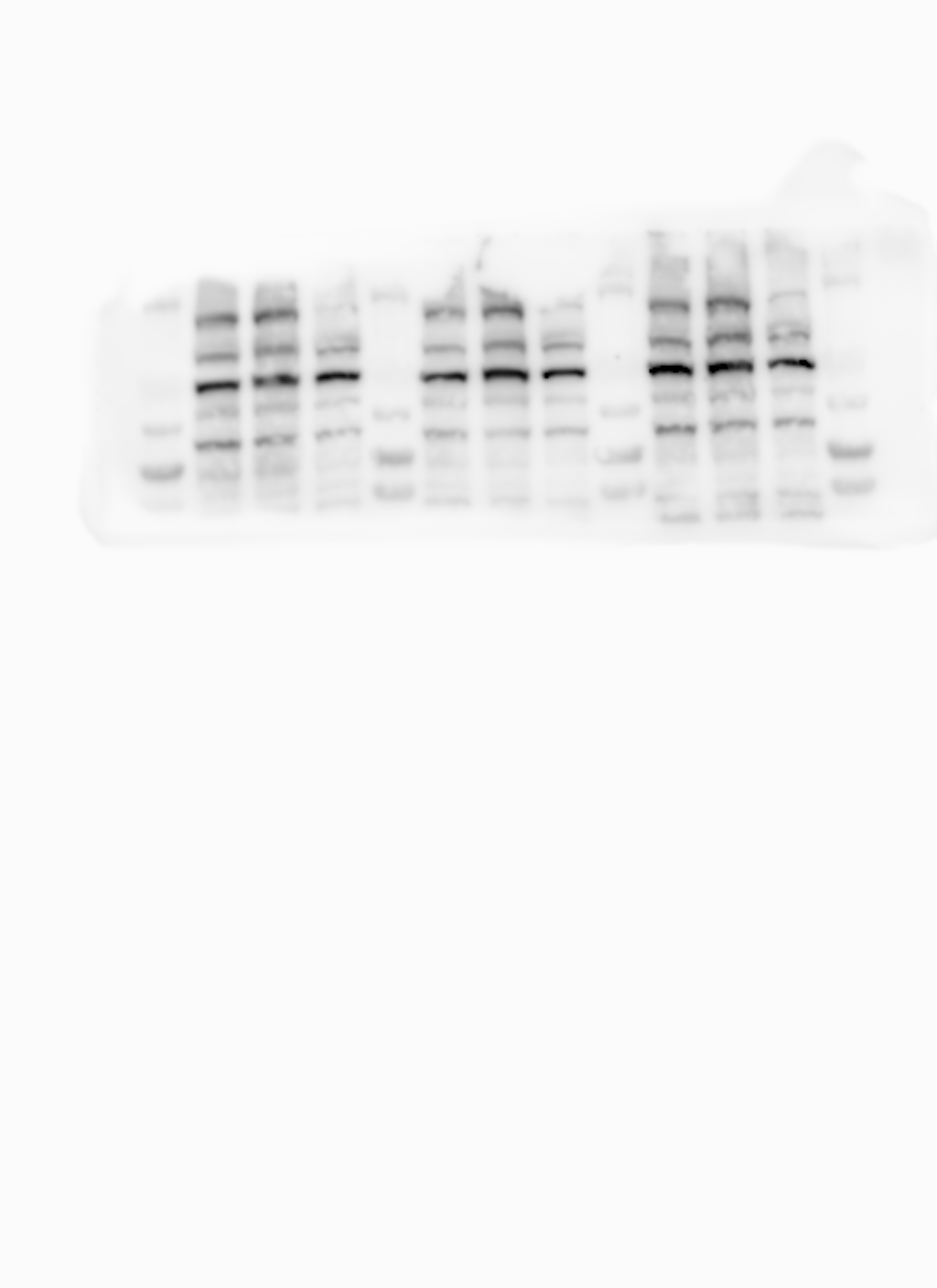

Supplement: Figure 5—source data 2. — Anti-mCh, anti-phosphorylated-Akt, and anti-total-Akt antibodies were used for the immunoblotting analysis. Alpha-tubulin was used as an internal control. [file elife-85293-fig5-data2.zip › Figure 5-source data 3.tif]

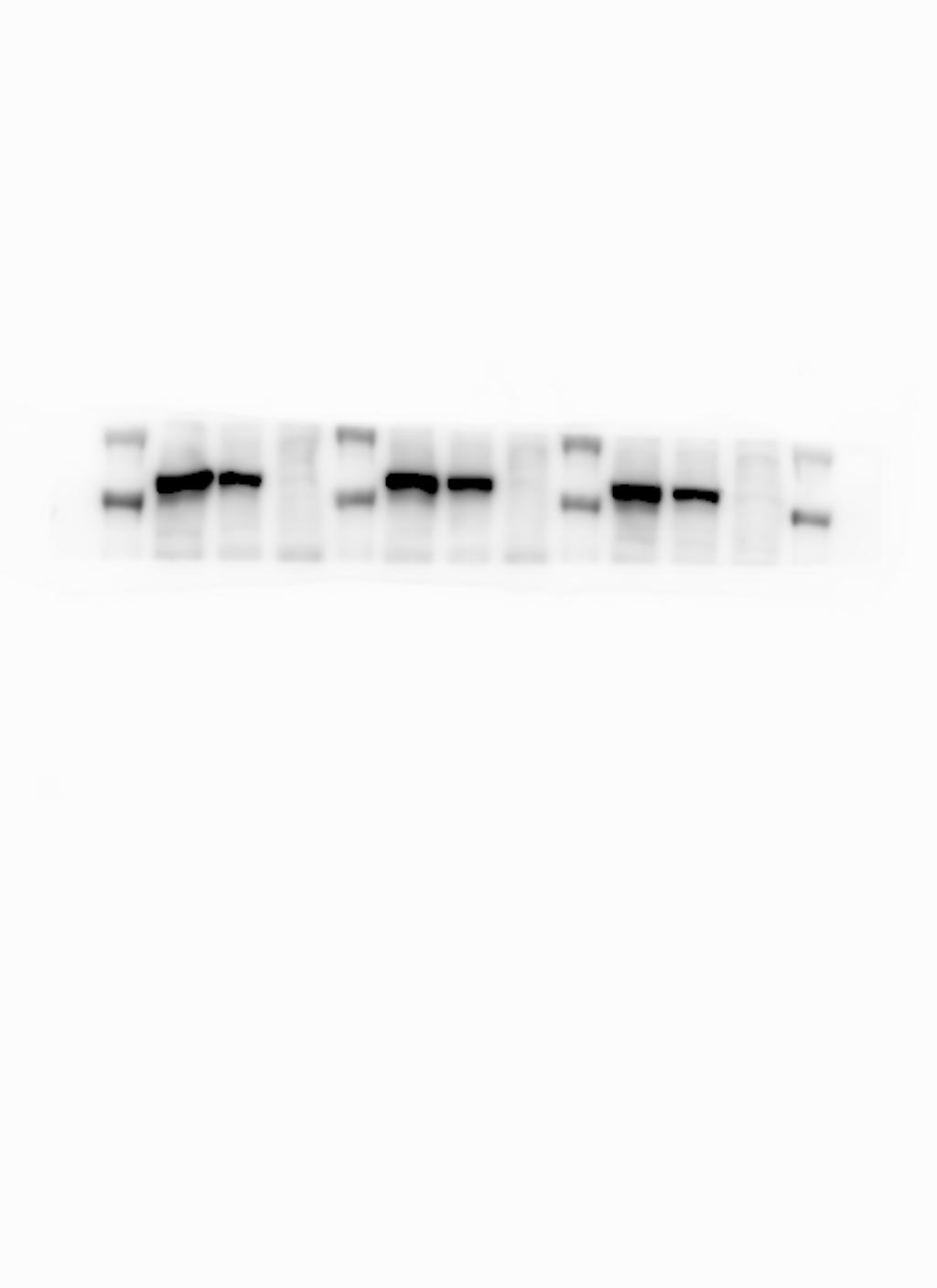

Supplement: Figure 5—source data 2. — Anti-mCh, anti-phosphorylated-Akt, and anti-total-Akt antibodies were used for the immunoblotting analysis. Alpha-tubulin was used as an internal control. [file elife-85293-fig5-data2.zip › Figure 5-source data 4.jpg]

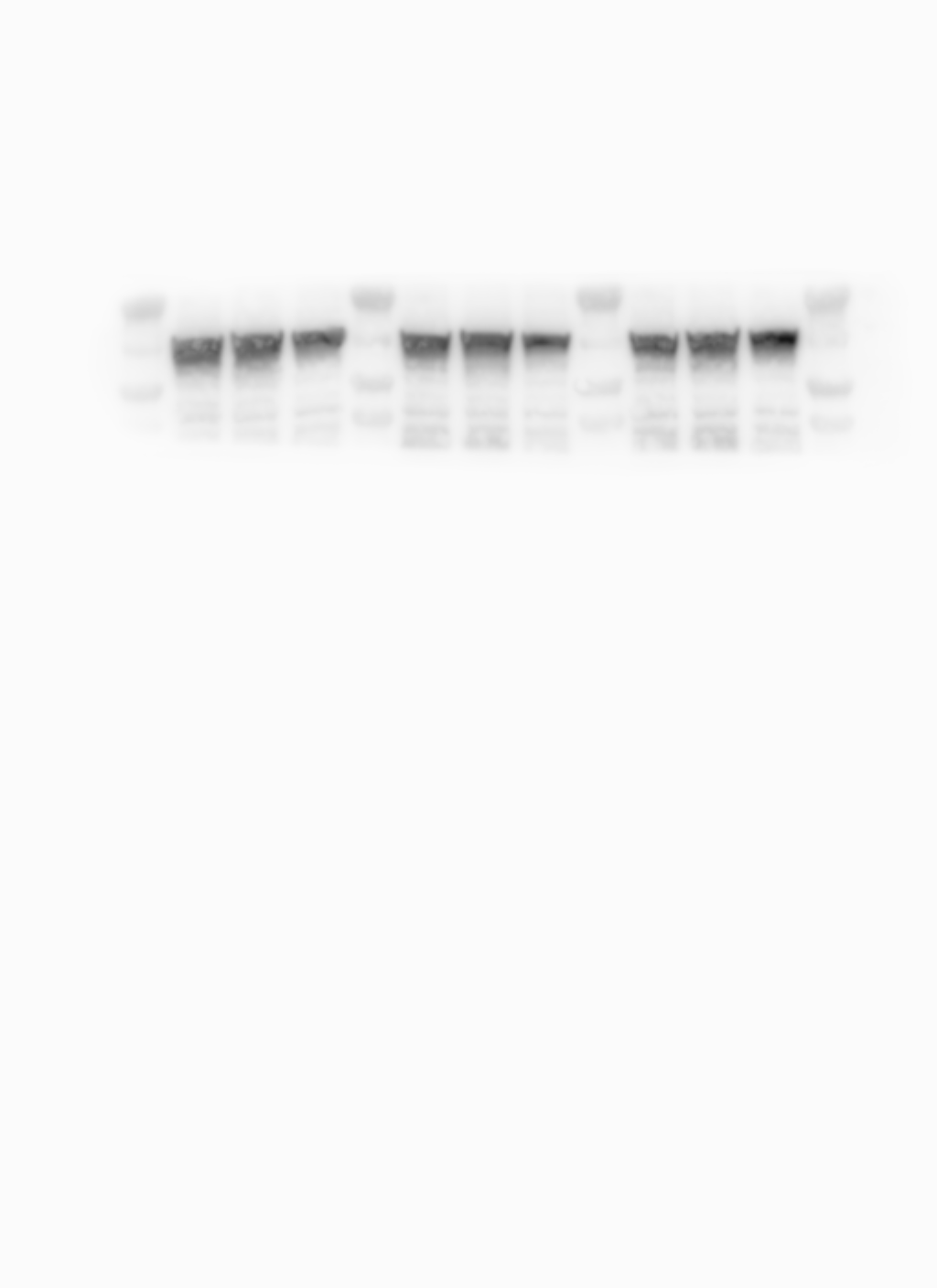

Supplement: Figure 5—source data 2. — Anti-mCh, anti-phosphorylated-Akt, and anti-total-Akt antibodies were used for the immunoblotting analysis. Alpha-tubulin was used as an internal control. [file elife-85293-fig5-data2.zip › Figure 5-source data 5.tif]

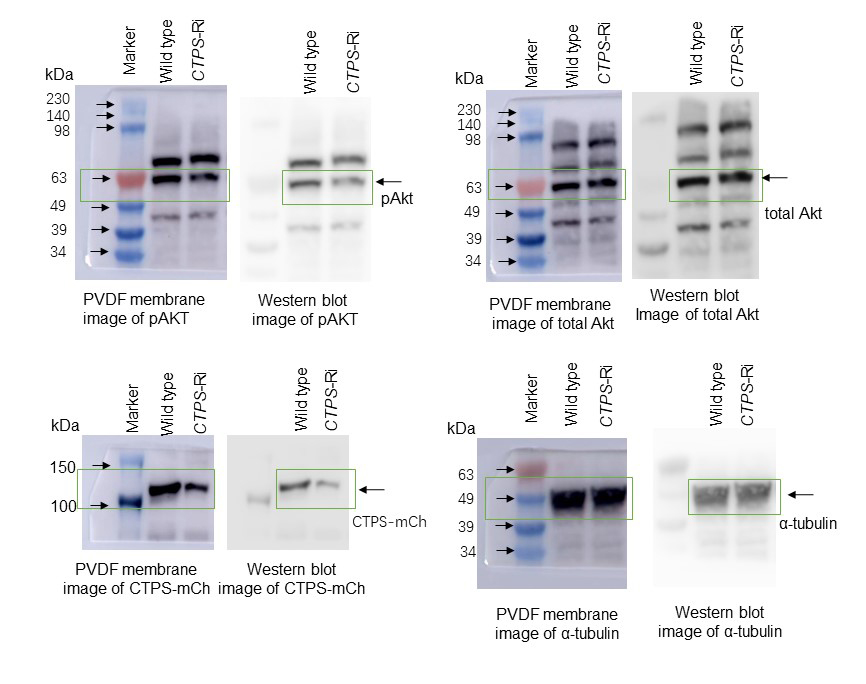

Supplement: Figure 5—source data 2. — Anti-mCh, anti-phosphorylated-Akt, and anti-total-Akt antibodies were used for the immunoblotting analysis. Alpha-tubulin was used as an internal control. [file elife-85293-fig5-data2.zip › Figure 5-source data 6.jpg]

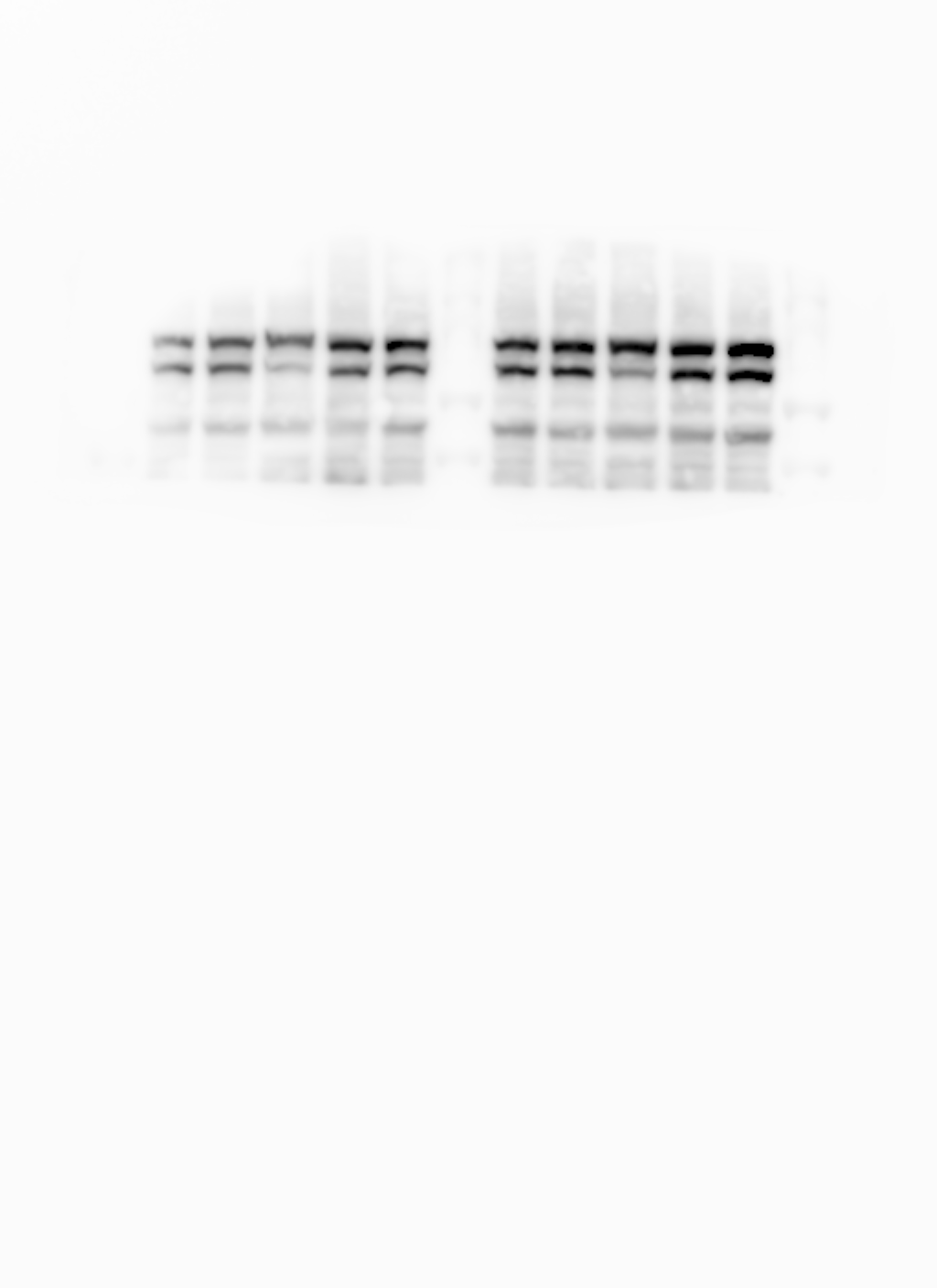

Supplement: Figure 6—source data 1. — Anti-mCh, anti-phosphorylated-Akt, and anti-total-Akt antibodies were used for the immunoblotting analysis. Alpha-tubulin was used as an internal control. [file elife-85293-fig6-data1.zip › Figure 6-source data 1.tif]

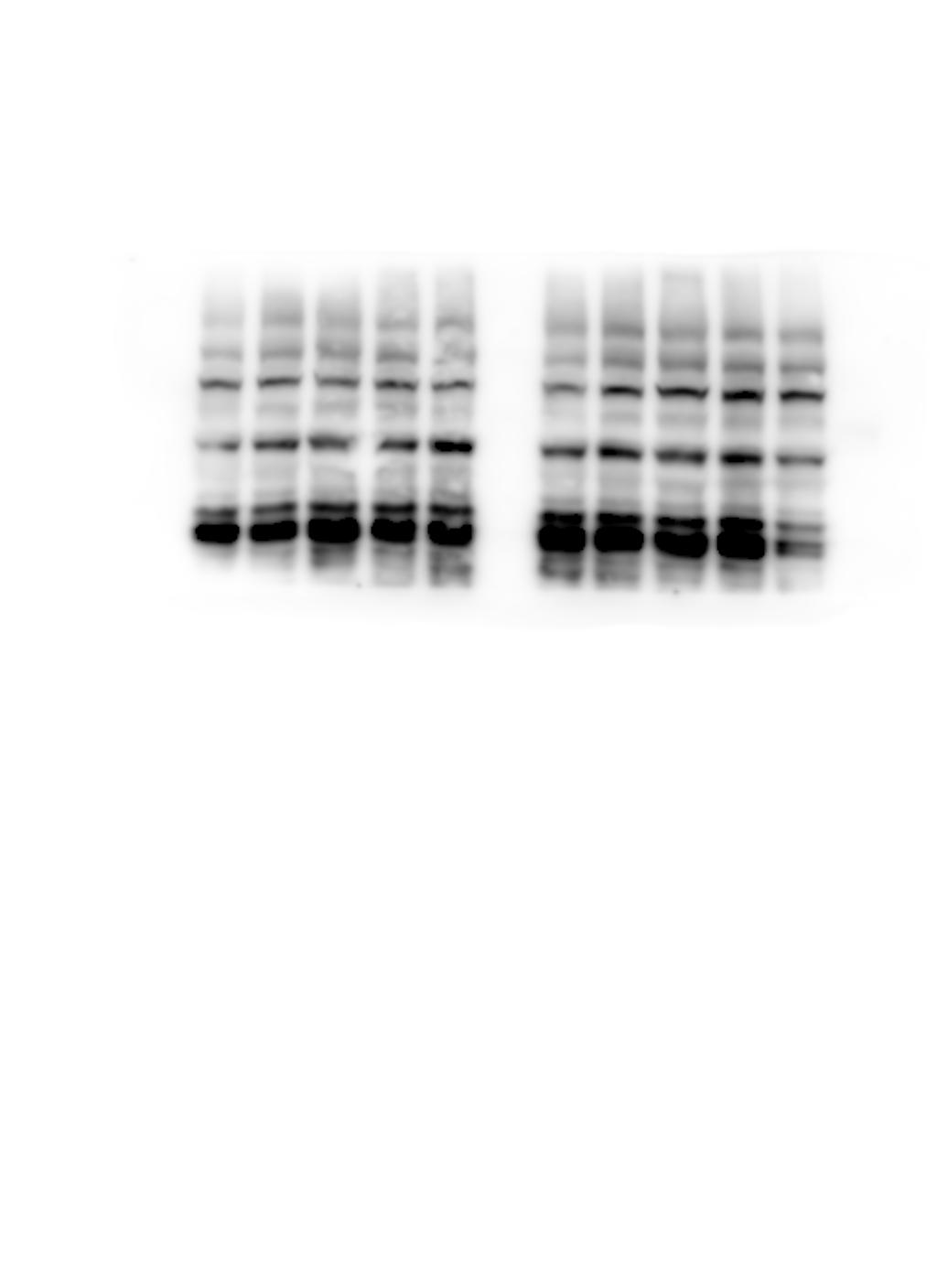

Supplement: Figure 6—source data 1. — Anti-mCh, anti-phosphorylated-Akt, and anti-total-Akt antibodies were used for the immunoblotting analysis. Alpha-tubulin was used as an internal control. [file elife-85293-fig6-data1.zip › Figure 6-source data 2.jpg]

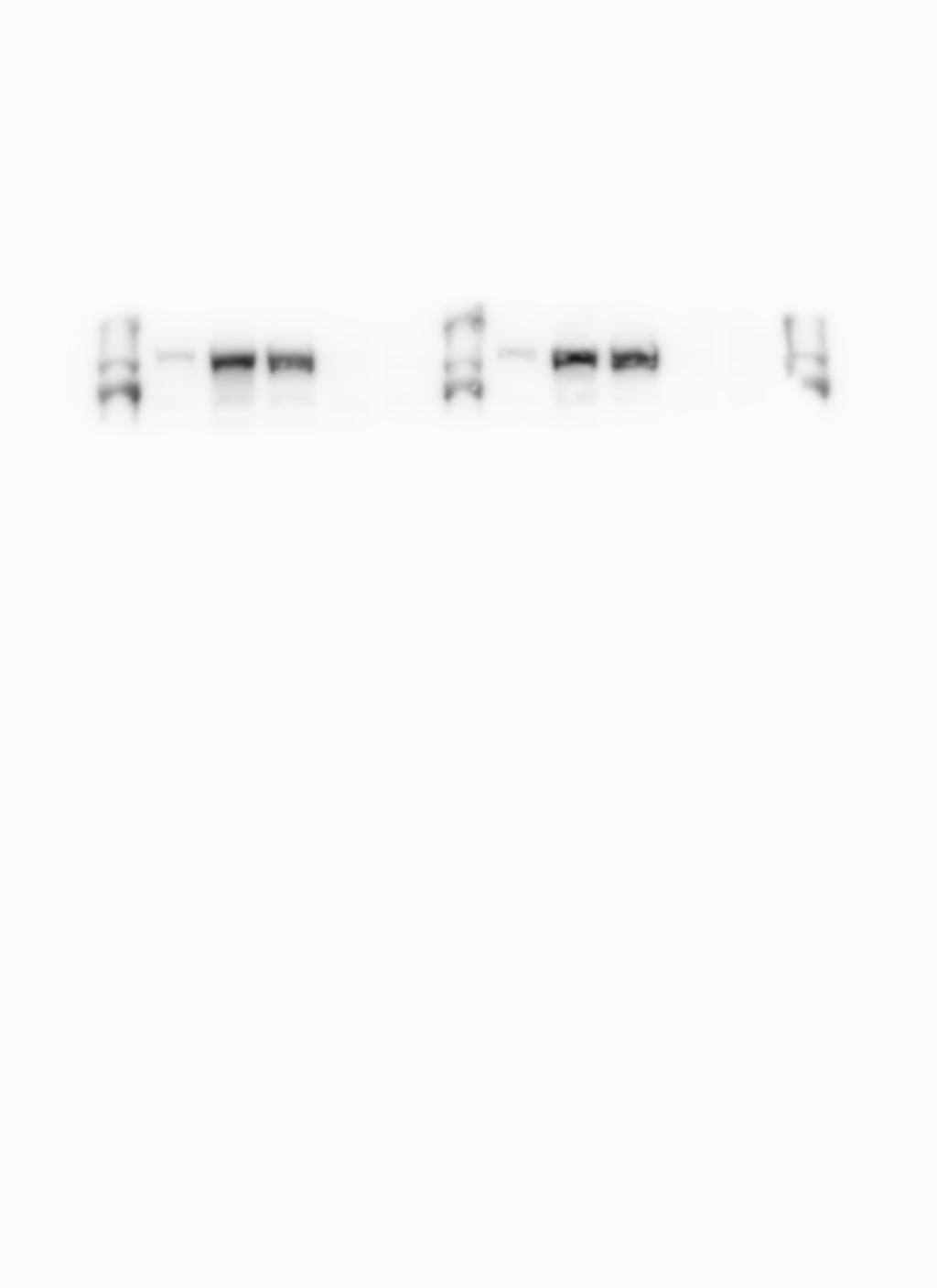

Supplement: Figure 6—source data 1. — Anti-mCh, anti-phosphorylated-Akt, and anti-total-Akt antibodies were used for the immunoblotting analysis. Alpha-tubulin was used as an internal control. [file elife-85293-fig6-data1.zip › Figure 6-source data 3.tif]

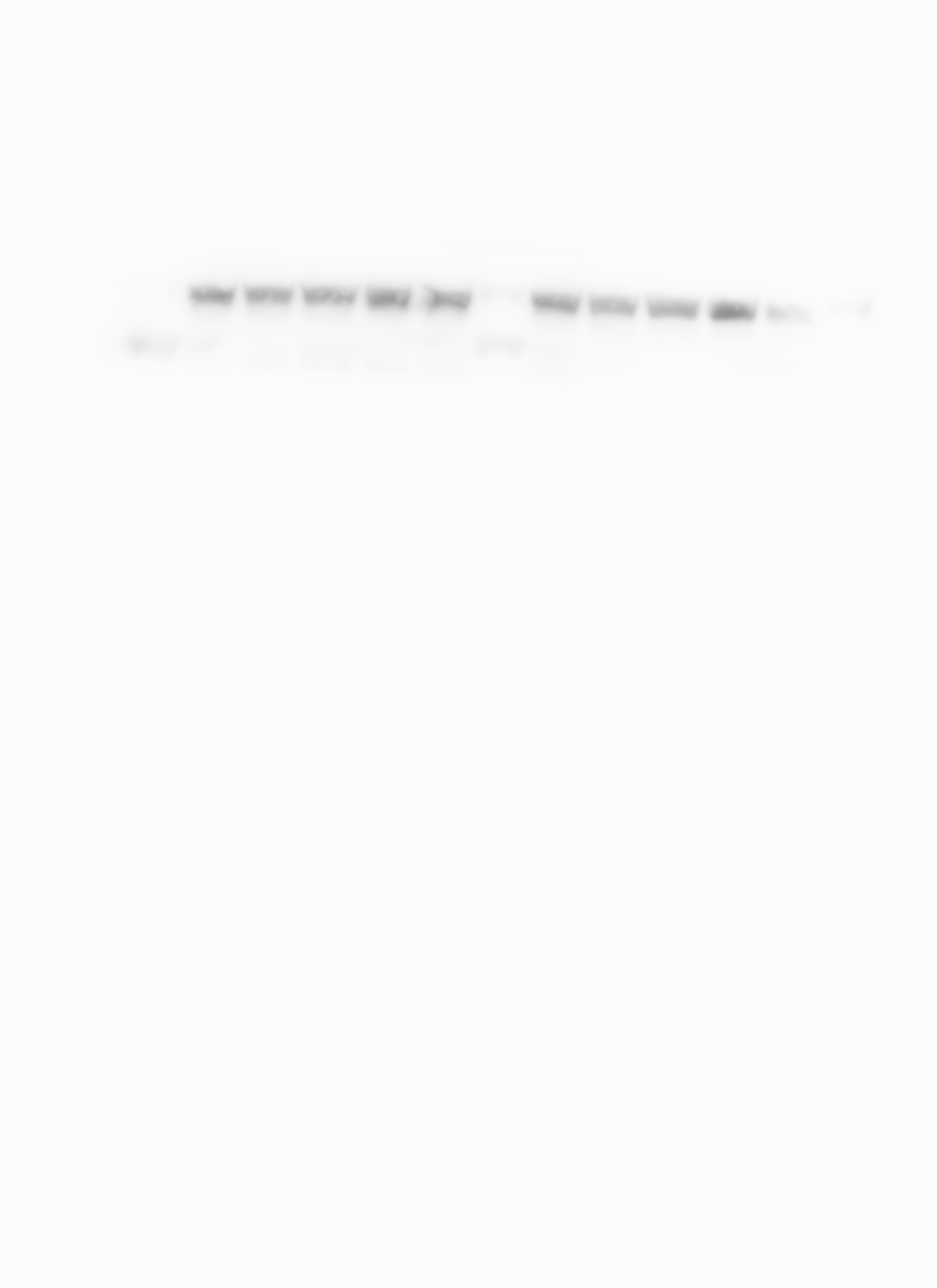

Supplement: Figure 6—source data 1. — Anti-mCh, anti-phosphorylated-Akt, and anti-total-Akt antibodies were used for the immunoblotting analysis. Alpha-tubulin was used as an internal control. [file elife-85293-fig6-data1.zip › Figure 6-source data 4.tif]

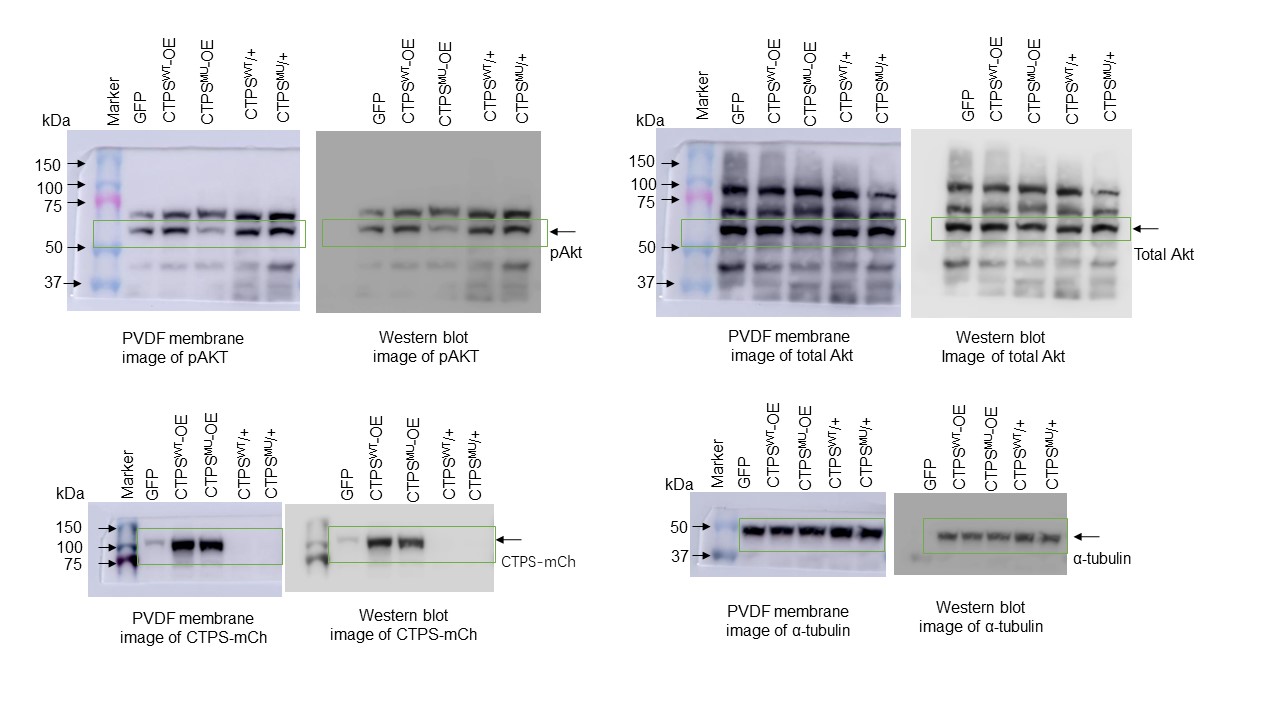

Supplement: Figure 6—source data 1. — Anti-mCh, anti-phosphorylated-Akt, and anti-total-Akt antibodies were used for the immunoblotting analysis. Alpha-tubulin was used as an internal control. [file elife-85293-fig6-data1.zip › Figure 6-source data 5.jpg]
